# Application of virtual microscopy in consultation practice of gastrointestinal and liver pathology

**DOI:** 10.4103/2153-3539.68333

**Published:** 2010-08-10

**Authors:** Shriram Jakate

**Affiliations:** Professor of Pathology, Gastroenterology and Hepatology, Director of Laboratory Services, Rush University Medical Center, Chicago, IL 60612, USA

## INTRODUCTION

For several years now, virtual microscopy has been utilized in medical teaching, research, proficiency testing, American Board of Pathology examinations, pathology meetings and conferences, and quality assurance programs. In diagnostic practice, it is most widely and routinely used in image analysis. Some practices are beginning to use it internally (within their group) for frozen section intraoperative consultations and subspecialty consultations due to geographic and time constrains.

Although diagnostic consultation for expert second opinion is a well-established practice in pathology for glass slides, similar consultation via virtual microscopy poses multiple controversial issues such as licensing, liability, security, reimbursement, and scanning quality and its validation. Regulations and standardization are not yet in place that pathologists can use to allay these fears. Nevertheless, if the virtual microscopy scans are of optimal quality, these can be simply substituted for glass slides and a microscope while maintaining all other practice guidelines for second opinion consultation. Additionally, the cost and maintenance of scanning equipment is not currently affordable to most practitioners and consultants.

A practice model that offers tertiary consultation in gastrointestinal (GI) and liver pathology is presented that eliminates the cost and maintenance of scanners by the consultants and clients, minimizes the need for review of glass slides, facilitates clinicopathological and radiological correlation and serves the need of clients who need timely help with challenging liver and GI cases and to obtain expert opinion for dysplasia in Barrett’s and ulcerative colitis surveillance biopsies required by the American Gastroenterology Association (AGA). This model has been practiced for 3 years and applied to over 2000 cases by small pathology practices in the 50 United States and a single consultant GI and liver pathologist.

## MATERIALS AND METHODS

A client (either solo practitioner pathologist or a small group of community hospital pathologists) encounters a GI or liver case that requires expert opinion and/or immunohistochemical stains. A preliminary pathology report is created and the following items are mailed (next day courier) to a large triage laboratory that serves as a secondary consultant: 1) requisition form filled with specific instructions for immunostains and specific questions for consultation, 2) Hemtoxylin and Eosin (H and E) -stained glass slide and a selected paraffin block pertaining to the specific questions/instructions, 3) clinically correlative materials such as endoscopy report, imaging report and laboratory data 4) patient demographic information and relevant prior pathology report.

The triage laboratory then performs the requested immunostains and additional immunostains, if needed, to address specific consultation questions. A new accession is created and all slides are scanned along with all reports and requisition. Pathologist(s) from the triage laboratory then attempt to address the specific questions regarding consultation. If it is felt that a tertiary consultation is required, a text message (and simultaneous e-mail) is then sent to the tertiary consultant who can access the entire case (demographic information, all scanned slides, all correlative reports and specific consultation questions both from the primary and secondary pathologists) on a protected website with a secure username and password. The tertiary consultant then reviews the case on the monitor with a large screen (24″) and high resolution (1920 × 1200 pixel resolution and 32 bit highest color quality), selects areas within the scanned whole slides for static images for the report, enters report and signs out the case accessioned by the triage laboratory. This creates an automatic alert to the primary pathologist, who can also review the case including selected areas and tertiary pathologist’s opinion by secure login on the same website. Most clients participate in a yearly survey for efficiency and quality of this triage consultation.

## RESULTS

The triage laboratory performs all routine and specialized immunostains and maintains several scanners and serves to bridge the gap between the primary and the consultant pathologist. The quality of scans is excellent with rare need for re-scanning. This triage consultation model serves the following purposes: 1) rapid turnaround time (average of 20 minutes after the consultant begins to review the case), 2) fulfill the need of expert consultation required by AGA as well as other challenging cases, 3) no need of owning or maintaining scanners by the primary or the consultant pathologist, 4) ability by the primary pathologist to immediately review the consultant pathologist’s report as well as selected areas within the scanned slides, 5) a rapid “glass-less” and “paper-less” reporting system, 6) flexibility for the consultant since cases can be reviewed anytime and anywhere, wherever Internet access is available.

The survey from primary pathologists endorses complete satisfaction by the pathologists as well as their respective clinical colleagues. Importantly, there has been no amendment of any report based on discordant opinion.

The need for “verification” or “validation” by re-review of glass slides is minimized due to the following: 1) excellent quality of scans and viewing ability, 2) slide(s) already reviewed by primary and secondary pathologists, 3) only a specific question is being addressed, 4) all clinically relevant correlative material is made available for review, 5) experience of 3 years and over 2000 cases, 6) endorsement of satisfactory service by the primary pathologists in their survey.

## CONCLUSION

The tertiary or “triage” consultation model has not been documented previously in the applications of virtual microscopy or whole slide scanning. This is highly effective for the specific needs of solo or small practice community hospital pathologists. Its track record needs to be considered in the potential regulations that are bound to be imposed in the future on virtual microscopy as a diagnostic technology.

